# Minimal residual disease by either flow cytometry or cytogenetics prior to an allogeneic hematopoietic stem cell transplant is associated with poor outcome in acute myeloid leukemia

**DOI:** 10.1038/s41408-017-0007-x

**Published:** 2017-11-27

**Authors:** Maxim Norkin, Lakshmikanth Katragadda, Fei Zou, Sican Xiong, Myron Chang, Yunfeng Dai, Jack W. Hsu, Jan S. Moreb, Helen Leather, Hemant S. Murthy, Nosha Farhadfar, Ying Li, Robert Hromas, Randy A. Brown, Christopher R. Cogle, John R. Wingard

**Affiliations:** 1 Division of Hematology and Oncology, Department of Internal Medicine, College of Medicine, University of Florida, Gainesville, FL USA; 2 Department of Biostatistics, College of Medicine, University of Florida, Gainesville, FL USA; 3 Department of Pathology, College of Medicine, University of Florida, Gainesville, FL USA

## Abstract

Relapsed acute myeloid leukemia (AML) is a significant challenge after allogeneic hematopoietic cell transplant (HCT). Multiparameter flow cytometry (MFC), conventional cytogenetics (CG), and fluorescence in situ hybridization (FISH) are routinely performed on bone marrow specimens prior to HCT to assess disease status. We questioned the extent by which pre-HCT evidence of minimal residual disease (MRD) detected by these standard assays, corresponded with post-HCT relapse. We conducted a single center, retrospective study of 166 AML patients who underwent HCT. Thirty-eight of one hundred sixty-six (23%) patients in complete remission (CR) or CR with incomplete count recovery (CRi) had MRD detectable by MFC, CG, or FISH. MRD was more frequently seen in patients with poor risk karyotype at diagnosis (*P* = 0.011). MRD-negative patients (MRD^neg^) had significantly longer overall survival (OS) and relapse-free survival than patients who were MRD positive (MRD^pos^) (*P* = 0.002 and 0.013, respectively). In patients with MRD^pos^ prior to HCT, the presence of acute graft vs. host disease (GVHD) (grade ≥ 2) or chronic GVHD significantly improved progression free survival (PFS) (hazard ratio (HR) = 0.053 (95% confidence interval (CI): 0.01–0.279), *P* = 0.0005) and OS (HR = 0.211 (95% CI: 0.081–0.547), *P* = 0.0014).

## Introduction

There have been only modest improvement in outcomes in acute myeloid leukemia (AML) over the last several decades. While most of this progress has come due to advances in supportive care, some benefit has resulted from better prognostication of AML and risk-adapted therapy^1, 2^. Approximately 60–80% of AML patients achieve CR after induction chemotherapy, although the majority eventually relapse due to surviving myeloblasts that are not detectable by light microscopic examination^3^. Using supplemental techniques such multiparameter flow cytometry (MFC), conventional cytogenetics (CG) and fluorescence in situ hybridization (FISH) we are able to detect the presence of low-level disease, commonly termed minimal residual disease (MRD). There is considerable interest in the impact of MRD status at the time of hematopoietic cell transplant (HCT) to predict post-HCT outcomes.

Several studies have reported adverse outcomes in patients who are MRD^pos^ prior to allogeneic HCT^4–6^. In these studies 10-color MFC was exclusively used for identifying MRD status. The significance of persistent abnormal karyotype as evidence of MRD is not as clear, with a limited number of studies with small sample size that produced contradictory results^7–13^. In addition, it remains unclear if intensification of the conditioning regimen and development graft-vs.-leukemia (GVL) effect accompanying graft vs. host disease (GVHD) are capable of mitigating the adverse impact of MRD on post-HCT outcomes.

In this study, we retrospectively evaluated the effect of pre-HCT MRD^pos^, by either 4-color MFC and/or CG/FISH on outcomes of AML patients undergoing HCT at our institution.

## Patients and methods

The study was approved by University of Florida institutional review board (UF IRB201400410). We retrospectively reviewed all AML patients aged ≥18 years in CR or CRi prior to HCT between January 2001 and January 2014. Surviving patients had at least 1 year of follow-up. AML disease status was assessed prior to HCT for all the patients by morphologic examination, 4-color MFC, CG, and FISH testing in bone marrow aspirate and biopsy specimens. CR was defined as bone marrow myeloblasts <5% by morphology in a normocellular bone marrow, absence of extra medullary leukemia, neutrophil count >1000/μL, and a platelet count >100,000/µL^14^. CRi was defined as meeting all the criteria for CR except for incomplete peripheral blood count recovery (neutrophils and/or platelets). Additional patient information, including time to relapse and mortality, were obtained from medical records, or from institutional database containing information regarding disease status, complications and survival through annual phone interviews.

Patient-specific, disease-specific, and HCT-specific variables known to impact AML prognosis were collected including^15^: age (<40 vs. 41–60 vs. >60 years); secondary AML diagnosis, defined as AML that developed after a history of antecedent hematologic disorder or after prior treatment with systemic chemotherapy and/or radiotherapy for a previous unrelated cancer; cytogenetic risk category (good, intermediate, poor) defined as per NCCN guidelines^16^; remission status at the time of HCT (first remission (CR1) vs. >CR1); achievement of CR vs. CRi; duration of CR1 (≤12 months vs. >12 months); intensity of HCT conditioning regimen^17^ (myeloablative vs. reduced intensity); donor type (matched sibling vs. others); and donor to recipient gender match (female donor-male recipient vs. other).

Assessment of MRD was performed on bone marrow aspirate samples obtained within 28 days prior to HCT using at least one of the following modalities: 4-color MFC, karyotyping utilizing CG, and FISH. Patients with evidence of disease by either technique were classified as being MRD^pos^, and patients who had absence of disease using these techniques, categorized as MRD^neg^.

MFC was performed on bone marrow specimens using monoclonal antibodies that were methodically used either as a large panel if the patient was newly evaluated or as a limited but targeted panel based on previously known patient-specific leukemia immune phenotype. MRD was reported as a percentage of CD45 positive white blood cells (WBCs) and was labeled MRD^pos^ if leukemic cells account for ≥0.1% of the analyzed total WBCs.

CG was performed using standard G-banding methods on 20 metaphase cells. FISH was reported as a percentage of abnormal nuclei among the examined 300 interphase nuclei. MRD^pos^ by CG was defined as abnormal karyotype seen in at least two metaphase cells, or less than two cells if it was a previously known abnormality for the given patient. FISH positivity of a prior known abnormality was labeled MRD^pos^. For our analysis, we combined the list of patients with MRD^pos^ by either CG or FISH.

As polymerase chain reaction (PCR) results for nucleophosmin (NPM1), fms-related tyrosine kinase 3 (FLT3), and CCAAT/enhancer binding protein alpha (CEBPA) mutations were not available for all patients, they were not used for assessment of MRD.

## Statistical analyses

Patient, disease, and transplant characteristics of MRD^pos^ and MRD^neg^ groups were compared by chi-square test and Wilcoxon rank-sum test, as appropriate. Relapse-free survival (RFS) was defined as time to relapse from HCT. Patients who are alive and continue to be in remission were censored for RFS at last day of contact. Patients who died without relapse were also censored for RFS at the day of death. We performed multivariate risk factor analysis by proportional sub-distribution hazards regression model. In risk factor analysis, RFS was used as the response variable. Patients who were not in CR or CRi at HCT were excluded for the analysis. Complete remission (CR vs. CRi) before HCT, karyotype risk category at diagnosis, duration of first remission (≤12 months vs. >12 months), CR status (CR1 vs. >CR1), conditioning regimen (myeloablative vs. others), age at HCT, gender, type of AML (de novo vs. secondary), donor type (matched sibling vs. other), and donor:recipient sex match (female donor:male recipient vs. all other gender combinations) were used as explanatory variables. A backward selection procedure at the 0.20 significance level was applied to form the final model including only important explanatory variables. Due to the nature of competing risk between relapse and death in HCT, we also estimated the distribution of RFS by the method of cumulative incidence rate. We compared distributions of RFS between patients with MRD^pos^ and MRD^neg^ by Gray test^18, 19^. Similarly, within MRD^pos^ patients, we compared distributions of RFS between those with and without acute and chronic GVHD using Gray test.

Overall survival (OS) was defined as time from HCT to death. Surviving patients were censored at date of last contact for OS. The Kaplan–Meier method was applied to estimate OS distribution. OS distributions between patients with MRD^pos^ and MRD^neg^ were compared by log-rank test. The multivariate Cox proportional hazards model and backward selection procedure were applied for the risk factor analysis with OS as the response variable and with the same explanatory variables as in the analysis for RFS. Within the cohort of MRD^pos^ patients we also compared distributions of OS between patients with and without acute and chronic GVHD using the Kaplan–Meier method. Data analyses were performed using R software^20^) and SAS software version 9.4 (SAS Institute Inc., Cary, NC, USA).

## Results

A total of 166 consecutive AML patients were identified as having CR/CRi prior to HCT and were included in the study. Baseline characteristics of patients are shown in Supplementary Table 1. The median follow-up among patients who were 46 months (range, 13–103).

Thirty-eight (23%) patients were MRD^pos^ by either MFC or CG/FISH. MRD was positive by MFC in 21 of 38 (55%) patients, by CG or FISH in 25 of 38 (66%) patients, and by both in 8 of 38 (21%) patients. Disease and HCT characteristics of the MRD^pos^ and MRD^neg^ groups are shown in Table 1. A significantly higher percentage of MRD^pos^ patients had a poor risk karyotype at the time of AML diagnosis (47% vs. 26%; *P* = 0.011), and had a trend toward having CRi at the time of HCT (26% vs. 14%; *P* = 0.077) in comparison to MRD^neg^ patients. The remaining variables known to be prognostic in AML were evenly balanced between the two groups.

**Table 1:** Comparison of pre-HCT variables between MRD^pos^ and MRD^neg^ cohorts

Covariate	Label	MRD + N (%)	MRD − N (%)	*P*-value
Total number (*N*)		38 (23)	128 (77)	
Age	<40	8 (21)	20 (16)	0.708
	40–59	20 (53)	69 (54)	
	≥60	10 (26)	39 (30)	
Gender	F	20 (53)	60 (47)	0.560
	M	18 (47)	67 (53)	
Cytogenetics	Favorable/intermediate risk	20 (53)	95 (74)	0.011
	Poor risk	18 (47)	33 (26)	
Transplant done in first remission (CR1)	CR1	28 (74)	97 (76)	0.792
	>CR1	10 (26)	31 (24)	
Transplant for relapsed AML: duration of CR1	>12 months	31 (82)	113 (88)	0.285
	≤12 months	7 (18)	15 (12)	
Secondary AML	No	23 (61)	78 (61)	0.964
	Yes	15 (39)	50 (39)	
Complete remission	CR	28 (74)	110 (86)	0.077
	CRi	10 (26)	18 (14)	
Conditioning regimen	Myeloablative	24 (63)	72 (56)	0.449
	Other	14 (37)	56 (44)	
Donor type	Matched sibling	12 (32)	42 (33)	0.887
	Other	26 (68)	86 (67)	
Female donor: male recipient (FDMR)	Other	28 (80)	91 (78)	0.844
	FDMR	7 (2)	25 (22)	

*MRD* minimal residual disease, *CR* complete remission, *CR1* first CR, *AML* acute myeloid leukemia, *CRi* CR with incomplete blood count recovery

### OS and RFS

**Fig. 1:**
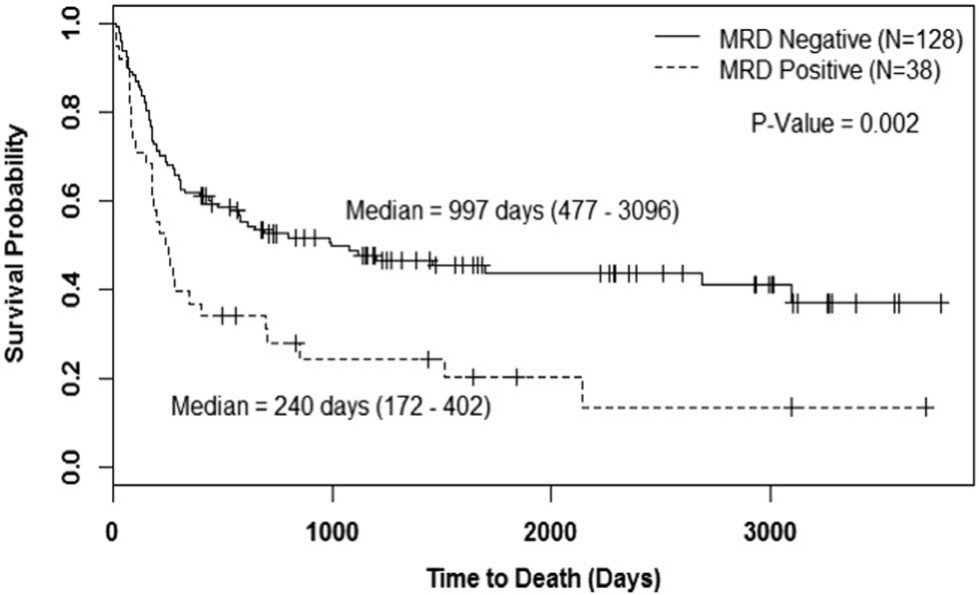
Overall survival with any MRD Overall survival based on any minimal residual disease (MRD) status irrespective of the type of MRD

OS curves for patients with MRD^pos^ and MRD^neg^ are presented in Fig. 1. Patients who were MRD^neg^ had significantly longer OS than patients who were MRD^pos^ (997 days (95% CI: 477–3096) vs. 240 days (95% CI: 172–402); *P* = 0.002). We performed a multivariate analysis using Cox proportional hazards model and backward selection procedure with OS as a response variable. The results revealed that patients who were MRD^neg^ (HR = 0.55 (95% CI: 0.35–0.87); *P* = 0.011), good or intermediate risk karyotype (HR = 0.51 (95% CI: 0.33–0.79), *P* = 0.003) and CR as compared to CRi (HR = 0.52 (95% CI: 0.31–0.87), *P* = 0.013) had significantly better OS (Table 2).

**Table 2:** Multivariate analysis of factors affecting OS^a^

Variable		HR	95% CI	*P*-value
MRD	Negative vs. positive	0.553	0.351–0.871	0.011
Karyotype risk	Good or intermediate vs. poor risk	0.512	0.331–0.791	0.003
Complete remission^b^	Yes vs. no	0.519	0.310–0.868	0.013
Secondary AML	No vs. yes	0.747	0.492–1.136	0.173

*AML* acute myeloid leukemia, *CI* confidence interval, *HR* hazard ratio, *MRD* minimal residual disease, *OS* overall survival

^a^Backward selection procedure was applied at the 0.2 significance level

^b^Complete remission vs. complete remission with incomplete count recovery

**Fig. 2:**
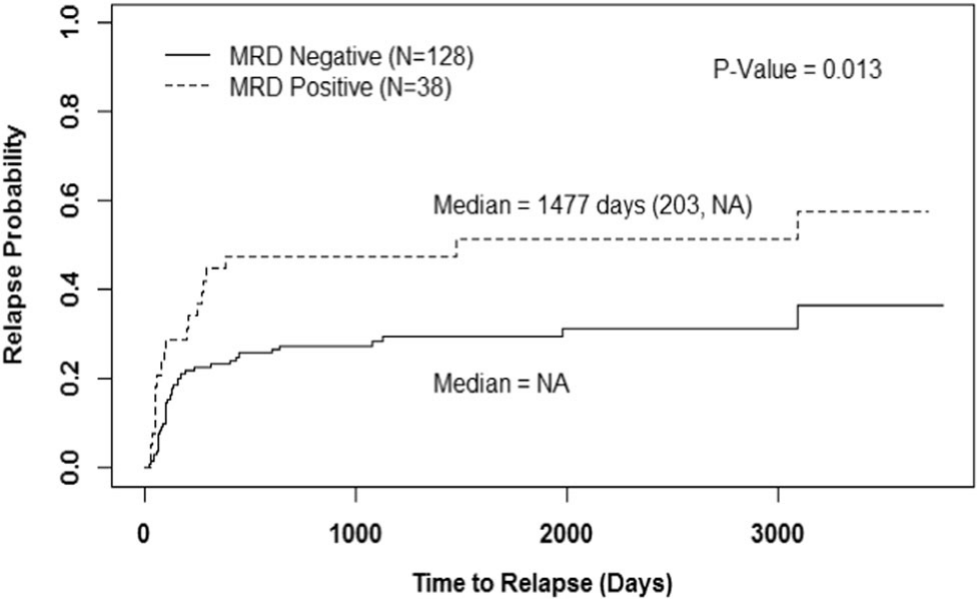
Cumulative incidence of relapse with any MRD Relapse-free survival based on any MRD status by method of cumulative incidence of relapse

The cumulative incidence of relapse for patients with MRD^pos^ and MRD^neg^ are shown in Fig. 2. Patients who were MRD^neg^ had significantly longer RFS than patients who were MRD^pos^ (*P* = 0.013). The median RFS was 1477 days for MRD^pos^ patients, and the median RFS in the MRD^neg^ group was not reached.

**Fig. 3:**
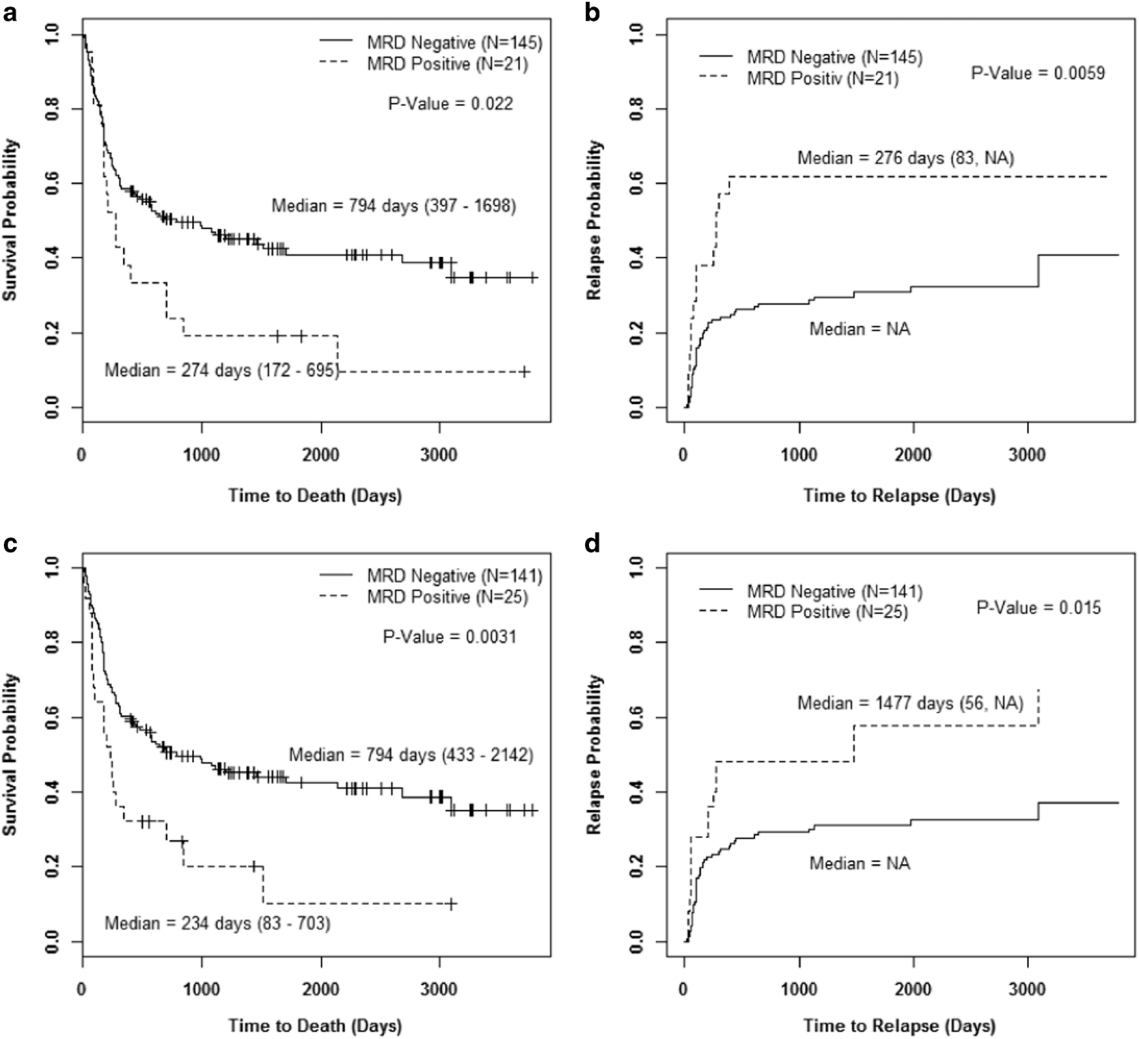
Overall survival and cumulative incidence of relapse based on MRD by flow or cytogenetics/FISH Overall survival and relapse-free survival by method of cumulative incidence of relapse assessed based on the type of minimal residual disease (MRD). **a** Overall survival based on MRD by flow. **b** Cumulative incidence of relapse based on MRD by flow. **c** Overall survival based on MRD by cytogenetics/FISH. **d** Cumulative incidence of relapse based on MRD by cytogenetics/FISH

In univariate analysis, MRD detected by either MFC or CG/FISH was associated with inferior OS (*P* = 0.022 and *P* = 0.0031, respectively; Fig. 3) and the type of assay used to detect MRD did not appear to affect the outcome. Therefore, we grouped the MRD^pos^ patients together for survival outcome assessment.

We performed a multivariate analysis using a proportional sub-distribution hazards regression model and backward selection with RFS as the response variable (Table 3). The analysis revealed that only good or intermediate risk karyotype was associated with significantly better RFS (HR = 0.49 (95% CI: 0.27–0.87); *P* = 0.016). MRD^neg^ patients trended toward better RFS (HR = 0.58 (95% CI: 0.32–1.08); *P* = 0.087). In univariate analysis, MRD detected by either MFC or CG/FISH was associated with shorter RFS (*P* = 0.0059 and *P* = 0.015, respectively) as seen in Fig. 3.

**Table 3:** Multivariate analysis of factors affecting RFS^a^

Variable		HR	95% CI	**P**-value
MRD	Negative vs. positive	0.584	0.316–1.081	0.087
Karyotype risk	Better or intermediate vs. poor risk	0.488	0.273–0.873	0.016
Donor status	Matched sibling donor vs. other	0.639	0.356–1.146	0.130
CR status at HCT	CR1 vs. >CR1	0.594	0.317–1.111	0.100
Secondary AML	No vs. yes	0.659	0.376–1.155	0.140
Conditioning regimen	Ablative vs. other	0.627	0.361–1.088	0.097

*AML* acute myeloid leukemia, *CI* confidence interval, *CR* complete response, *HCT* hematopoietic stem cell transplantation, *HR* hazard ratio, *MRD* minimal residual disease, *RFS* relapse-free survival

^a^Backward selection procedure was applied at the 0.2 significance level

### Non-relapse mortality

**Fig. 4:**
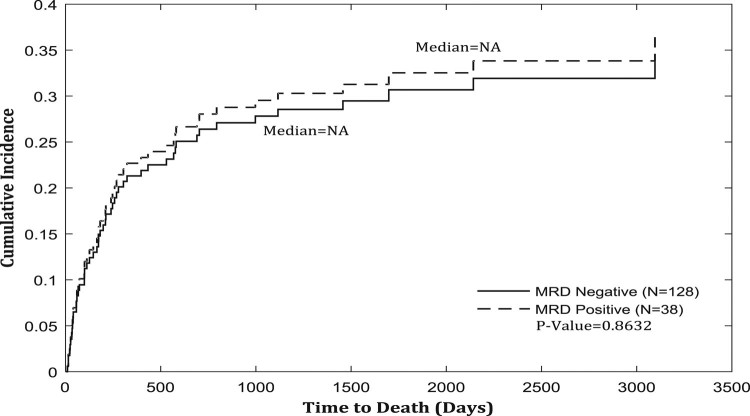
Non-relapse mortality based on MRD status by method of cumulative incidence

There was no difference in the cumulative incidence of non-relapse mortality between MRD^pos^ and MRD^neg^ groups (*P* = 0.86) as shown in Fig. 4.

### Analysis of MRD^pos^ patients

Within the MRD^pos^ patient population, we compared those who survived more than 1 year to those who lived 1 year or less. Patient who lived longer than a year were less likely to have poor risk karyotype at baseline (29% vs. 58%), more often received a matched sibling donor graft (50% vs. 25%), less often had MRD detected by persistent CG/FISH abnormalities (64% vs. 75%), less often received an ablative conditioning regimen (29% vs. 42%) and most importantly had a higher incidence of acute and chronic GVHD (86% vs. 58%). Based on these observations we analyzed GVHD in MRD^pos^ patients.

### Effect of GVHD among MRD^pos^ patients

Results from the multivariate Cox proportional hazards model and backward selection procedure with RFS as the response variable are presented in Table 4a. The multivariate analysis revealed that the following variables were significantly associated with RFS: MRD by CG or FISH (HR = 0.051 (95% CI: 0.009–0.297); *P* = 0.0009), MRD by MFC (HR = 0.15 (95% CI: 0.04–0.569), *P* = 0.0053), the presence of acute GVHD (Grade ≥ 2) or chronic GVHD (HR = 0.053 (95% CI: 0.01–0.279), *P* = 0.0005).

**Table 4:** Multivariate analysis of factors affecting RFS^a^ (a) and OS^a^ (b) within MRD^pos^ patients

**(**a**)**				
Variable		HR	95% CI	**P**-value
Both acute (grade ≥ 2) or chronic GVHD	Yes vs. no	0.053	0.01–0.279	0.0005
MRD by karyotype or FISH	Negative cytogenetics or FISH vs. positive cytogenetics or FISH	0.051	0.009–0.297	0.0009
MRD by flow cytometry	Negative vs. positive	0.15	0.04–0.569	0.0053
Remission status at HCT	CR vs. CRi	5.824	0.639–53.107	0.1182
Duration of CR	≥12 months vs. <12 months	0.333	0.068–1.633	0.1753
Intensity of conditioning	Ablative vs. others	2.497	0.665–9.375	0.1753

**(b)**				
Variable		HR	95% CI	**P**-value
Type of CR	CR vs. CRi	0.12	0.035–0.414	0.0008
Both acute (grade ≥ 2) or chronic GVHD	Yes vs. No	0.211	0.081–0.547	0.0014
CR status at HCT	CR1 vs. >CR1	5.564	1.671–18.527	0.0052
Donor type	MSD vs. Other	0.257	0.077–0.862	0.0277
Karyotype Risk	Better or Intermediate vs. Poor Risk	0.426	0.175–1.034	0.0593
MRD by flow cytometry	Negative vs. Positive	0.493	0.173–1.405	0.1855

*CI* confidence interval, *CR* complete remission, *CRi* CR with incomplete blood count recovery, *FISH* fluorescence in situ hybridization, *GVHD* graft vs. host disease, *HR* hazard ratio, *MRD* minimal residual disease, *RFS* relapse- free survival, *HCT* hematopoietic stem cell transplantation, *MSD* matched sibling donor

^a^Backward selection procedure was applied at the 0.2 significance level

The development of acute GVHD, chronic GVHD, either acute or chronic GVHD, and acute and chronic GVHD significantly improved OS (*P* = 0.02, *P* = 0.0003, *P* = 0.03, and *P* = 0.0001, respectively). Results from the multivariate Cox proportional hazards model and backward selection procedure with OS as response variable are presented in Table 4b. CR status at HCT (HR = 5.564 (95% CI: 1.671–18.527); *P* = 0.0052), CR vs. CRi (HR = 0.12 (95% CI: 0.035–0.414); *P* = 0.0008), both acute (grade ≥ 2) and chronic GVHD (HR = 0.211 (95% CI: 0.081–0.547); *P* = 0.0014), and donor status (HR = 0.257, (95% CI: 0.077–0.862); *P* = 0.0277) were significantly associated with OS.

## Discussion

In this study, we demonstrated that the presence of MRD by MFC, CG, or FISH prior to HCT predicted inferior OS and RFS in AML patients. We also showed that the development of acute and chronic GVHD mitigated the adverse impact of detectable MRD on RFS and OS in these patients. Early studies have shown that evidence of MRD by flow cytometry after achieving a “morphological CR” predicts for an increased relapse rate and shorter RFS in AML patients^21^. Subsequent MRD studies post induction^22, 23^ or post consolidation therapy confirmed poor RFS and OS in multivariate analysis irrespective of age^24, 25^. Poor AML outcomes were also reported in patients with evidence of MRD by residual abnormal karyotype while in morphological remission^23^. Several recent retrospective single institution studies demonstrated the negative impact of MRD^pos^, assessed by 10-color MFC, prior to HCT on the probability of relapse in AML patients^4–6^. In one study, the MRD^pos^ prior to HCT was as predictive of post-HCT relapse as the presence of active disease^4^. The significance of MRD assessed by CG or FISH on AML outcomes after HCT is not as clear. The published reports are limited in number and sample size, and have reported contradictory findings, with some reporting worse outcomes^10, 8, 13^, and others reporting no significant difference^5, 11^. Based on these reports, MRD by MFC appears to be clearly predictive of poor outcomes but the significance of karyotype needed further clarification.

In our study, we evaluated pre-HCT MRD by MFC and CG/FISH. When assessed separately on univariate analysis, MRD^pos^ status by either MFC or CG/FISH was associated with significantly worse RFS and OS. As outcomes did not differ by the method of MRD assessment and given the relatively small sample size, we combined all patients with any type of MRD positivity. Patients who were MRD^neg^ prior to HCT, by either MFC or CG/FISH, had significantly better OS and a trend toward better RFS on multivariate analysis. Our results suggest that evidence of MRD before HCT, regardless of the modality of testing used, predicts for poor AML outcomes.

It is well known that development of GVHD is associated with lower relapse rates in leukemia post HCT, due to the GVL effect^26^. However, MRD^pos^ prior to HCT is still associated with very high relapse risk and inferior survival despite the development of GVHD. It is still unclear whether the GVL effect observed in patients with acute or chronic GVHD is capable of overcoming higher relapse rates observed in AML patients who are MRD^pos^ prior to HCT. Here we report a very important observation that the development of acute and chronic GVHD, and presumably its accompanying GVL effect, may overcome the adverse effect of MRD^pos^, at least in some patients.

The retrospective nature of our study has some limitations. As expected, more than half of our patients had a normal karyotype at diagnosis. MRD in these patients was therefore only evaluated by MFC, as we did not have access to PCR testing for MRD in many patients, who were referred from outside hospitals. Although there are emerging data being accumulated about the prognostic significance of numerous molecular markers these tests are still not routinely utilized for MRD measurement in AML patients prior to HCT. In this study, we used only commonly accepted techniques for MRD monitoring such as MFC, CG, and FISH. The relatively small sample size did not allow us to analyze the statistical significance of residual disease detected separately by MFC or CG/FISH in a multivariate analysis. Although small, our study has the second largest group of patients (*n* = 25) with residual CG/FISH. It will be useful to know the threshold of MRD (if one exists) above which the prognosis of AML is adversely affected. While earlier studies attempted to identify this threshold there is no consensus on this issue at this time^24, 25^. This could not be elucidated in our report as well.

Prior studies have reported improved outcomes in patients with MRD^pos^ ^5, 7, 24, 27^ who proceed to HCT, but our study shows the prognosis of this subset of patients to be generally poor after HCT in comparison to patients with no MRD. This is in line with recent publications^4–6^. Importantly, we identified that development of acute or chronic GHVD can mitigate adverse effect of MRD^pos^ on survival. In can be explained that GVL effect associated with development of acute and chronic GVHD can successfully prevent disease relapse in some patents with MRD^pos^. In agreement with prior reports, our study also showed that the intensity of the conditioning regimen did not have major effect on outcomes of MRD^pos^ patients^5, 28^. Larger studies may help discern other differences among MRD^pos^ patients with better survival vs. poorer survival and may also clarify if MRD has an additive role when present with other known poor prognostic risk factors.

## Conclusion

MRD^pos^ status prior to HCT, identified by either MFC, CG, or FISH, correlates with shortened survival times after HCT. MRD^pos^ patients continue to have poor prognosis compared to MRD^neg^ patients despite HCT, although development of acute or chronic GVHD appears to have a positive impact on RFS and OS of MRD^pos^ patients compared to MRD^pos^ patients who do not develop acute or chronic GVHD.

## Electronic supplementary material

Supplemental Table 1
