# Supplementary material for: Minimal residual disease by either flow cytometry or cytogenetics prior to an allogeneic hematopoietic stem cell transplant is associated with poor outcome in acute myeloid leukemia
Source: Blood Cancer J. 2017 Nov 27;7(12):634. doi: 10.1038/s41408-017-0007-x (PMC5802525; doi:10.1038/s41408-017-0007-x)
Supplement: Supplementary file 1 — Supplemental Table 1 [file 41408_2017_7_MOESM1_ESM.docx]

| **Patient and Disease Characteristics** | **Number (%) [range]** |
| --- | --- |
| Eligible AML patients in CR or CRi | 166 |
| Male : Female ratio | 1.07 |
| Median age, years | 53 [19 - 74] |
| Age, yrs < 40 / 40-59 / ≥60 | 28 (16) / 89 (54) / 49 (30) |
| Favorable risk cytogenetics | 11(7) |
| Intermediate risk cytogenetics | 104 (63) |
| Poor risk cytogenetics | 51 (30) |
| HCT in CR1 | 125 (75) |
| HCT for AML in CR2 or CR3 = (>CR1 ) | 37+ 4 = 41 (25) |
| HCT for AML in CR2 or CR3 in which ,CR1 ≤ 12 months | 22 (13) |
| Secondary AML | 65 (39) |
| HCT in CRi | 29 (17) |
| Median follow-up for surviving patients, months | 46 [13 - 103] |
|  |  |
| **Transplant Characteristics** | **Number (%)** |
| Myeloablative regimen* | 96 (58) |
| Other regimens | 70 (42) |
| FDMR (female donor male recipient) | 32 (19) |
| Matched sibling donor | 55 (33) |
| Matched unrelated donor | 94 (57) |
| Haploidentical donor | 14 (8) |
| Umbilical cord donor | 3 (2) |
|  |  |
| **MRD characteristics** | **Number (%)** |
| Patients with any MRD^pos^ before HCT | 38 (23) |
| MRD^pos^ by cytogenetics or FISH only | 25 (15) |
| MRD^pos^ by MFC only | 21 (13) |
| MRD^pos^ by both cytogenetics and MFC | 8 (5) |
| AML- Acute myeloid leukemia , CR- complete remission , CRi- CR with incomplete blood count recovery , CR1- first CR , CR2- second CR , CR3- third CR , HCT- allogeneic hematopoietic transplant , MRD- Minimal residual disease , * Ref: Bacigalupo et al BBMT 2009 | |

Supplemental Table 1. Baseline Patient and Disease Characteristics
